# Premature Senescence and Cardiovascular Disease Following Cancer Treatments: Mitochondria-Mediated Mechanistic Insights

**DOI:** 10.3390/ijms272110011

**Published:** 2026-11-08

**Authors:** Sudhir Pandey

**Affiliations:** Department of Cosmeceutics, College of Pharmacy, China Medical University, Shui-Nan Campus, No. 100, Sec.1 Jingmao Road, Beitun District, Taichung 406040, Taiwan; pandeysudhir523@gmail.com

**Keywords:** cardio-oncology, cellular senescence, cardiovascular disease, mitochondrial dysfunction, SASP, anthracyclines, radiotherapy, cancer therapeutics

## Abstract

The remarkable enhancement in cancer survival attained over recent decades has shifted attention toward the long-term cardiovascular effects of anticancer therapies. Beyond overt cardiotoxicity, accumulating evidence indicates that many conventional chemotherapeutics, including radiotherapy, targeted agents, and emerging immunotherapies, accelerate biological aging of the cardiovascular system through the induction of premature cellular senescence. Senescent endothelial cells, cardiomyocytes, vascular smooth muscle cells, cardiac fibroblasts, and resident immune cells exhibit persistent growth arrest accompanied by metabolic reprogramming, chronic inflammation, mitochondrial dysfunction, and acquisition of a senescence-associated secretory phenotype (SASP), collectively contributing to adverse cardiac remodeling, vascular dysfunction, myocardial fibrosis, and accelerated atherosclerosis. Among the interconnected mechanisms driving therapy-induced senescence, mitochondria have emerged as central regulators integrating oxidative stress, DNA damage responses, bioenergetic alterations, impaired mitophagy, mitochondrial dynamics, calcium dysregulation, and innate immune activation. Through the production of reactive oxygen species (ROS), chronic inflammatory signaling, the release of mitochondrial DNA, and the activation of inflammasomes, dysfunctional mitochondria actively amplify senescence rather than acting only as organelles that produce energy. This creates self-sustaining feed-forward loops that promote cardiovascular aging long after cancer treatment is completed. This review critically examines the molecular mechanisms linking anticancer therapies to premature cardiovascular senescence with particular emphasis on mitochondria-centered signaling networks. I address therapy-specific mitochondrial alterations induced by HER2-targeted treatments, immune checkpoint inhibitors, ionizing radiation, platinum-based drugs, anthracyclines, and more recent anticancer modalities, emphasizing interconnected pathways despite disparate fundamental mechanisms of action. Emerging biomarkers of mitochondrial dysfunction and senescence, together with therapeutic prospects involving senolytics, senomorphics, mitochondria-targeted antioxidants, metabolic modulators, and interventions reestablishing mitochondrial quality control, are also evaluated. Finally, I identify unresolved questions limiting clinical translation and propose future research directions aimed at preserving cardiovascular health in the rapidly expanding population of cancer survivors.

## 1. Introduction

Advances in early cancer diagnosis, precision medicine, molecularly targeted therapies, immunotherapy, and optimized multimodal treatment approaches have transformed the prognosis of many malignancies, resulting in a steadily expanding population of long-term cancer survivors. While this therapeutic triumph denotes a major accomplishment in modern medicine, it has also uncovered a growing burden of chronic treatment-related complications that extend well beyond tumor eradication. Cardiovascular disease (CVD) has erupted as one of the foremost non-malignant causes of morbidity and mortality among cancer survivors, frequently resembling or even exceeding the risk of recurrent malignancy in selected patient populations. Consequently, cardio-oncology has evolved into a rapidly developing interdisciplinary field dedicated to understanding, preventing, and managing cardiovascular complications associated with cancer and its treatment [1,2].

Historically, cardiotoxicity has been viewed primarily through the lens of acute myocardial injury, left ventricular dysfunction, arrhythmias, or heart failure. However, increasing experimental and clinical evidence advocates that cardiovascular toxicity incorporates a broader spectrum of biological alterations that resemble accelerated aging rather than isolated tissue injury [3]. Patients receiving anthracyclines, radiotherapy, platinum compounds, HER2-targeted agents, vascular endothelial growth factor inhibitors, proteasome inhibitors, or immune checkpoint inhibitors are at elevated risk of long-term cardiovascular complications. These complications comprise persistent vascular dysfunction, myocardial fibrosis, impaired cardiac reserve, arterial stiffness, endothelial dysfunction, and premature atherosclerosis, which can persist or emerge years to decades after completion of cancer therapy [4,5]. These long-lasting pathological changes cannot be fully explained by direct cytotoxicity alone, indicating the involvement of sustained maladaptive cellular programs.

Among these programs, cellular senescence has gained considerable attention as a unifying mechanism linking cancer therapy to chronic cardiovascular dysfunction. Cellular senescence is characterized by irreversible withdrawal from the cell cycle accompanied by profound phenotypic remodeling rather than passive cellular inactivity. The cardiovascular system appears particularly vulnerable to cancer therapy-induced senescence because several resident cell populations—including endothelial cells, cardiomyocytes, vascular smooth muscle cells, cardiac fibroblasts, pericytes, and immune cells—display limited regenerative capacity and remain metabolically active throughout life [6]. Persistent senescence within these populations contributes to endothelial dysfunction, microvascular rarefaction, impaired angiogenesis, myocardial stiffness, ventricular remodeling, electrical instability, and accelerated vascular aging. Notably, these pathological processes often evolve silently over prolonged periods before clinical manifestations become evident, accentuating the need to understand their initiating molecular events [4,7].

Mitochondria have emerged as central orchestrators of this process, and their strategic integration into multiple cellular stress-response pathways makes them particularly susceptible to the diverse insults generated by anticancer therapies. Anthracyclines directly interfere with mitochondrial topoisomerase IIβ and promote excessive reactive oxygen species (ROS) generation; ionizing radiation induces persistent oxidative stress and mitochondrial DNA damage; platinum compounds disrupt mitochondrial genome integrity; targeted therapies alter metabolic signaling; and immune checkpoint inhibitors indirectly enhance inflammatory mitochondrial stress through immune activation. Although these therapies differ substantially in their primary molecular targets, they converge on remarkably similar mitochondrial abnormalities [5].

Mitochondrial dysfunction initiates a complex cascade involving excessive ROS production, impaired electron transport chain activity, mitochondrial DNA damage, defective mitochondrial biogenesis, dysregulated fusion–fission dynamics, insufficient mitophagy, altered NAD^+^ metabolism, and persistent activation of DNA damage response pathways [8]. These abnormalities reinforce one another through self-perpetuating feedback loops, ultimately stabilizing the senescent phenotype. Moreover, damaged mitochondria actively communicate with the nucleus and immune system through mitochondrial danger-associated molecular patterns, including oxidized mitochondrial DNA, cardiolipin exposure, and mitochondrial RNA, thereby activating cyclic GMP–AMP synthase–stimulator of interferon genes (cGAS–STING), nucleotide-binding oligomerization domain-like receptor family pyrin domain-containing 3 (NLRP3) inflammasome signaling, and nuclear factor-κB (NF-κB)-dependent inflammatory pathways. Such mechanisms position mitochondria not merely as passive victims of therapy-induced damage but as active drivers of chronic cardiovascular remodeling [9,10].

Growing recognition of mitochondria-mediated senescence has also expanded therapeutic opportunities. Strategies aimed at preserving mitochondrial quality control, enhancing mitophagy, restoring mitochondrial biogenesis, reducing mitochondrial ROS production, replenishing NAD^+^ pools, modulating mitochondrial dynamics, and selectively eliminating senescent cells have demonstrated encouraging results in preclinical models of therapy-induced cardiotoxicity. Nevertheless, significant challenges remain before these interventions can be translated into routine clinical practice. The temporal evolution of senescence, tissue-specific mitochondrial adaptations, interactions with aging and metabolic comorbidities, and identification of reliable biomarkers for early detection remain incompletely understood.

Furthermore, preserving cardiovascular function must not compromise the antitumor efficacy of cancer therapies, necessitating carefully balanced therapeutic strategies. Although previous reviews have comprehensively described cancer therapy-induced cardiovascular senescence and aging, the specific contribution of mitochondrial quality control failure as a central mechanistic driver remains insufficiently addressed [3,11]. In this review, we present a comprehensive and mitochondria-centered perspective on premature cardiovascular senescence induced by contemporary cancer treatments. We first summarize the biological foundations of therapy-induced senescence and discuss how distinct anticancer modalities converge on shared mitochondrial stress pathways. We then critically evaluate the roles of mitochondrial oxidative stress, DNA damage, mitophagy, mitochondrial dynamics, metabolic remodeling, and inflammatory signaling in promoting long-term cardiovascular disease. Finally, we examine emerging biomarkers and therapeutic approaches targeting mitochondrial dysfunction and senescence while outlining key knowledge gaps that should guide future translational and clinical investigations. By integrating mechanistic insights across molecular, cellular, and clinical levels, this review aims to provide a unified framework for understanding how mitochondrial dysfunction links cancer therapy to accelerated cardiovascular aging and to identify novel opportunities for cardioprotection in cancer survivors.

## 2. Mitochondrial Reactive Oxygen Species and DNA Damage as Central Drivers of Therapy-Induced Cardiovascular Senescence

Mitochondria occupy a central position in the pathogenesis of cancer therapy-associated cardiovascular disease by integrating oxidative metabolism, cellular stress responses, and inflammatory signaling. While physiological levels of mitochondrial reactive oxygen species (mtROS) function as essential second messengers regulating cellular adaptation, persistent oxidative stress following anticancer treatment disrupts mitochondrial integrity and initiates molecular events that culminate in premature cardiovascular senescence [12,13,14]. Rather than representing a passive consequence of mitochondrial injury, excessive mtROS establishes self-amplifying feedback circuits involving DNA damage, defective mitochondrial quality control, metabolic remodeling, and chronic inflammatory activation. These interconnected mechanisms are increasingly recognized as common biological denominators underlying delayed cardiovascular complications after diverse cancer treatments [15,16].

### 2.1. Mitochondria as the Principal Source of Persistent Oxidative Stress

The mitochondrial electron transport chain (ETC) generates ATP through oxidative phosphorylation while simultaneously producing controlled amounts of ROS. Under normal conditions, electron leakage from complexes I and III generates superoxide anions that are detoxified by mitochondrial antioxidant systems, including manganese superoxide dismutase (MnSOD/SOD2), glutathione peroxidases, peroxiredoxins, and thioredoxin-dependent pathways [17]. This balance allows mitochondria to participate in redox signaling without causing macromolecular damage.

Cancer therapies disrupt this redox equilibrium through multiple mechanisms. Anthracyclines, particularly doxorubicin, accumulate within cardiac mitochondria due to their interaction with cardiolipin, an inner mitochondrial membrane phospholipid essential for respiratory chain organization [18]. Doxorubicin-mediated disruption of mitochondrial electron transport promotes excessive ROS generation, while inhibition of mitochondrial topoisomerase IIβ compromises transcription of mitochondrial respiratory genes, further impairing oxidative phosphorylation efficiency [19]. Because cardiomyocytes possess high mitochondrial density and depend extensively on oxidative metabolism, they are particularly susceptible to persistent mitochondrial oxidative injury.

Ionizing radiation induces both immediate and delayed mitochondrial oxidative stress. Although radiation initially generates free radicals through radiolysis of intracellular water molecules, long-term mitochondrial dysfunction persists through damage to respiratory chain proteins, membrane lipids, and mitochondrial DNA (mtDNA) [20,21]. This sustained mitochondrial ROS production provides a mechanistic explanation for delayed radiation-associated cardiovascular disease, including accelerated vascular aging, fibrosis, and coronary pathology. Similarly, platinum-based chemotherapy, targeted kinase inhibitors, and immune-based therapies can indirectly induce mitochondrial stress by altering calcium homeostasis, metabolic signaling, inflammatory pathways, and mitochondrial turnover [22,23,24,25]. Despite differences in their primary pharmacological targets, many anticancer treatments converge on mitochondrial ROS accumulation as a shared mechanism promoting cardiovascular aging.

### 2.2. Oxidative DNA Damage and Activation of Senescence Pathways

Persistent mtROS damages both mitochondrial and nuclear genomes. Oxidative DNA lesions, including 8-oxo-7,8-dihydroguanine (8-oxoG), single-strand breaks, and double-strand DNA breaks, accumulate when antioxidant defenses and repair mechanisms become insufficient [26,27]. Nuclear DNA damage activates the canonical DNA damage response (DDR), involving ATM, ATR, checkpoint kinases, and stabilization of p53. Activated p53 promotes transcription of cyclin-dependent kinase inhibitors such as p21^Cip1^, resulting in durable cell-cycle arrest [28,29]. A parallel senescence pathway involves activation of the p16^Ink4a^-retinoblastoma (Rb) axis, which reinforces irreversible growth arrest independently of p53 [30]. Persistent activation of these pathways converts transient stress responses into stable senescent states characterized by altered metabolism, impaired tissue repair capacity, and inflammatory secretion.

In the cardiovascular system, this process has major pathological consequences because many cardiac cell populations exhibit limited regenerative potential. Cardiomyocytes exposed to oxidative stress accumulate DNA damage and mitochondrial dysfunction, while endothelial cells, vascular smooth muscle cells, and cardiac fibroblasts develop senescence-associated phenotypes that impair vascular integrity and myocardial remodeling [31,32,33,34]. Cancer therapy-induced senescence-like phenotype therefore represents a form of accelerated cardiovascular biological aging rather than a simple acute toxic response. Because adult cardiomyocytes are terminally differentiated and have limited proliferative capacity, cancer therapy-induced senescence in the heart is more appropriately interpreted as a senescence-like stress-adaptation state characterized by persistent DNA damage signaling, mitochondrial dysfunction, inflammatory activation, and impaired cellular homeostasis rather than classical proliferative arrest.

### 2.3. Mitochondrial DNA Damage: A Persistent Molecular Memory of Cancer Therapy

mtDNA is particularly vulnerable to oxidative injury because of its proximity to the ETC, limited protective histone packaging, and relatively restricted repair capacity compared with nuclear DNA [35]. Human mtDNA encodes essential components of oxidative phosphorylation complexes; therefore, mtDNA damage directly compromises mitochondrial bioenergetics and enhances respiratory inefficiency. Following cancer therapy exposure, mtDNA mutations, deletions, and reduced copy number can persist long after treatment completion. Anthracycline exposure has been associated with reduced mitochondrial respiratory capacity and impaired expression of mitochondrially encoded proteins, whereas radiation promotes mtDNA oxidative lesions and genomic instability [36,37,38,39]. These abnormalities create a self-reinforcing cycle whereby defective oxidative phosphorylation increases electron leakage, further elevating ROS production and accelerating mitochondrial genome damage.

Loss of mitochondrial integrity also disrupts mitochondrial biogenesis. Reduced activity of regulators such as peroxisome proliferator-activated receptor gamma coactivator-1α (PGC-1α), nuclear respiratory factors (NRF1/NRF2), and mitochondrial transcription factor A (TFAM) limits replacement of damaged mitochondria and promotes accumulation of dysfunctional organelles [40,41,42]. In cardiomyocytes, this impaired mitochondrial renewal contributes to energetic failure, contractile dysfunction, and susceptibility to heart failure.

### 2.4. Mitochondrial ROS Links Senescence to the SASP

The pathological importance of mtROS extends beyond DNA damage because mitochondria actively regulate inflammatory signaling associated with senescence. Persistent oxidative stress activates redox-sensitive transcription factors including nuclear factor-κB (NF-κB), activator protein-1 (AP-1), and CCAAT/enhancer-binding proteins (C/EBPs), promoting expression of inflammatory mediators collectively known as the senescence-associated secretory phenotype (SASP) [43,44].

In cardiovascular tissues, SASP factors including interleukin-6 (IL-6), IL-1β, tumor necrosis factor-α (TNF-α), monocyte chemoattractant protein-1 (MCP-1), and transforming growth factor-β (TGF-β) promote endothelial dysfunction, immune cell recruitment, fibroblast activation, and extracellular matrix remodeling [45,46]. Senescent endothelial cells exhibit reduced nitric oxide bioavailability and impaired angiogenic capacity, whereas senescent cardiac fibroblasts contribute to myocardial fibrosis and ventricular stiffening. Importantly, mitochondria are both regulators and targets of SASP signaling. Inflammatory cytokines released from senescent cells can further increase mitochondrial ROS generation through activation of NADPH oxidases, mitochondrial calcium overload, and suppression of antioxidant pathways, establishing a positive-feedback loop that maintains chronic cardiovascular inflammation [46].

### 2.5. Mitochondria as Inflammatory Hubs Linking Cancer Therapy to Cardiovascular Senescence

A major conceptual advance in cardiovascular senescence biology is the recognition that damaged mitochondria function as inflammatory signaling platforms. Dysfunctional mitochondria release damage-associated molecular patterns (DAMPs), particularly oxidized mtDNA, cardiolipin, and mitochondrial RNA, which activate innate immune pathways [44,47]. Cytosolic mtDNA is sensed by cyclic GMP–AMP synthase (cGAS), leading to activation of stimulator of interferon genes (STING), TANK-binding kinase 1 (TBK1), interferon regulatory factor 3 (IRF3), and NF-κB signaling [44,48]. This pathway promotes transcription of inflammatory cytokines and reinforces SASP development. In endothelial cells and vascular tissues, chronic cGAS–STING activation contributes to inflammatory remodeling and vascular dysfunction.

Additionally, mtROS and oxidized mtDNA activate the NLRP3 inflammasome, resulting in caspase-1 activation and maturation of IL-1β and IL-18. These cytokines promote myocardial fibrosis, endothelial activation, and adverse ventricular remodeling. Thus, mitochondrial damage provides a mechanistic bridge between cancer therapy exposure, cellular senescence, sterile inflammation, and cardiovascular disease.

### 2.6. Therapeutic Implications: Targeting Mitochondrial Oxidative Stress

**Figure 1:**
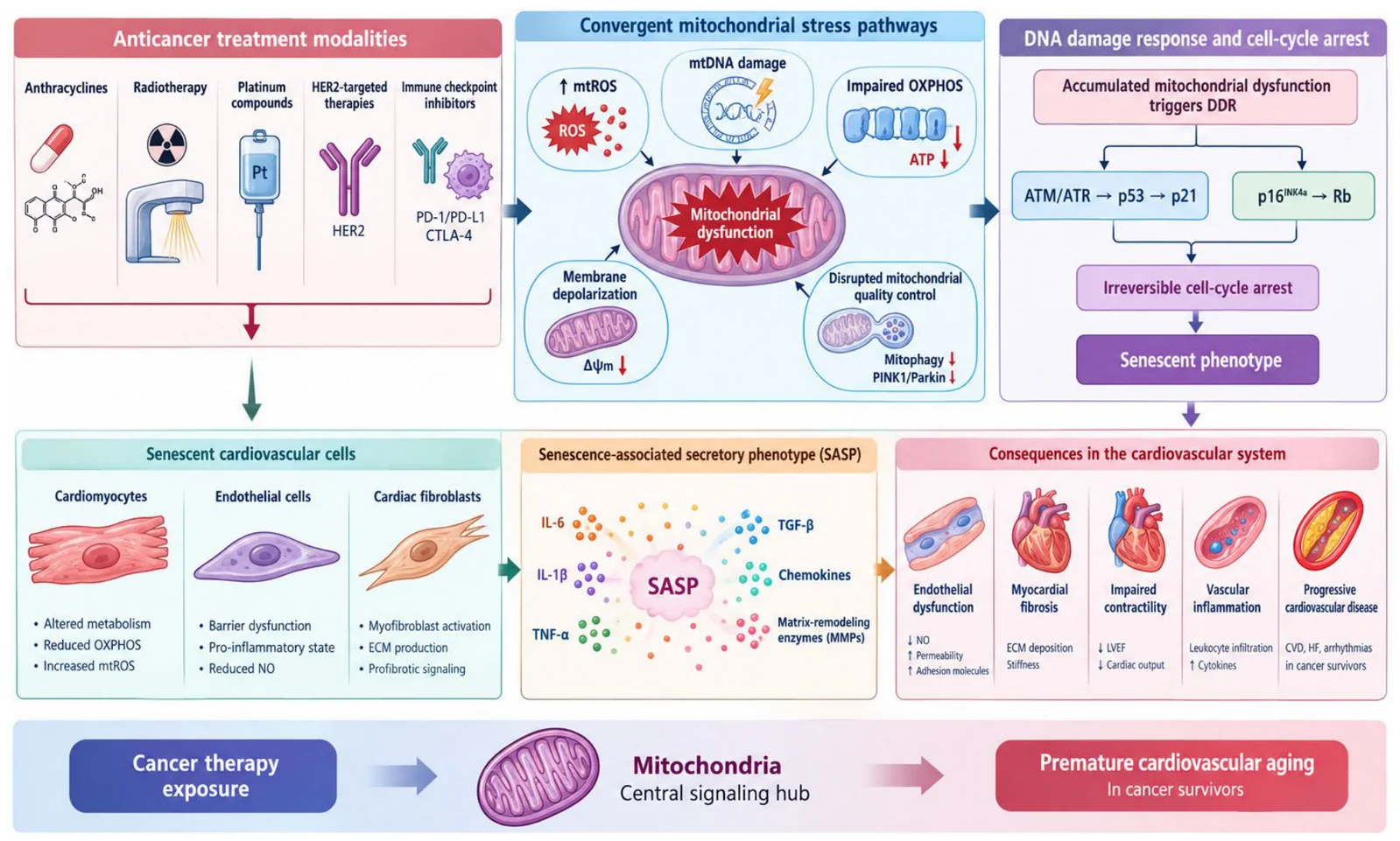
Cancer therapy-induced mitochondrial dysfunction drives premature cardiovascular senescence. Major anticancer treatment modalities induce convergent mitochondrial stress pathways that accelerate cardiovascular biological aging. Anthracyclines, radiotherapy, platinum compounds, HER2-targeted therapies, and immune checkpoint inhibitors promote mitochondrial reactive oxygen species (mtROS) generation, mitochondrial DNA (mtDNA) damage, impaired oxidative phosphorylation (OXPHOS), mitochondrial membrane depolarization, and disruption of mitochondrial quality control. Accumulation of dysfunctional mitochondria activates DNA damage response (DDR) pathways, including ATM/ATR–p53–p21 and p16^Ink4a^-Rb signaling, resulting in irreversible cell-cycle arrest and acquisition of a senescent phenotype. Senescent cardiomyocytes, endothelial cells, and cardiac fibroblasts exhibit altered metabolism and release a senescence-associated secretory phenotype (SASP) enriched in IL-6, IL-1β, TNF-α, TGF-β, chemokines, and matrix-remodeling enzymes. These processes promote endothelial dysfunction, myocardial fibrosis, impaired contractility, vascular inflammation, and progressive cardiovascular disease in cancer survivors. The figure illustrates mitochondria as a central signaling hub linking cancer therapy exposure to premature cardiovascular aging. Abbreviations: ATM, ataxia telangiectasia mutated; ATR, ATM and Rad3-related kinase; DDR, DNA damage response; HER2, human epidermal growth factor receptor 2; IL, interleukin; mtDNA, mitochondrial DNA; mtROS, mitochondrial reactive oxygen species; OXPHOS, oxidative phosphorylation; SASP, senescence-associated secretory phenotype; TGF-β, transforming growth factor beta; TNF-α, tumor necrosis factor alpha.

Understanding mtROS as a central driver of therapy-induced cardiovascular senescence has stimulated interest in mitochondria-directed cardioprotective strategies. Conventional systemic antioxidants have shown limited clinical benefit because indiscriminate ROS suppression may interfere with physiological redox signaling and anticancer mechanisms. Consequently, current approaches focus on selective modulation of mitochondrial oxidative stress and quality control pathways. Mitochondria-targeted antioxidants such as mitoquinone (MitoQ), Szeto–Schiller peptides, and related compounds have demonstrated protective effects against oxidative mitochondrial injury in experimental cardiovascular models [49]. Restoration of mitochondrial turnover through enhanced mitophagy, activation of PGC-1α-dependent mitochondrial biogenesis, and correction of NAD^+^ depletion represent additional strategies to improve mitochondrial resilience [50]. Future therapeutic approaches will likely require combination strategies integrating mitochondrial protection, senescent cell modulation, and inflammatory pathway inhibition while preserving anticancer efficacy (Figure 1). Identification of circulating mitochondrial biomarkers, including mtDNA fragments, oxidative damage products, and SASP-associated molecules, may enable earlier detection of cardiovascular injury and facilitate personalized cardio-oncology interventions.

## 3. Mitochondrial Quality Control Failure: Mitophagy Impairment and Mitochondrial Dysfunction in Cancer Therapy-Induced Cardiovascular Senescence

Mitochondrial integrity is maintained through a highly coordinated mitochondrial quality control (MQC) network responsible for identifying, repairing, recycling, and replacing dysfunctional mitochondria. These processes include mitochondrial proteostasis, selective mitochondrial autophagy (mitophagy), mitochondrial fusion and fission dynamics, mitochondrial biogenesis, and metabolic adaptation. Under physiological conditions, MQC preserves mitochondrial fitness by eliminating damaged organelles and maintaining cellular energy homeostasis. This regulation is particularly critical in cardiovascular tissues because cardiomyocytes, endothelial cells, and vascular smooth muscle cells rely heavily on mitochondrial metabolism and possess limited regenerative capacity [51].

Cancer therapies disrupt multiple components of MQC simultaneously by increasing mitochondrial injury while impairing repair mechanisms. As a result, defective mitochondrial surveillance promotes accumulation of damaged mitochondria, excessive ROS production, impaired ATP generation, and activation of senescence-associated inflammatory pathways. Increasing evidence indicates that MQC failure represents a convergent mechanism linking diverse anticancer treatments to delayed cardiovascular dysfunction and premature cardiovascular aging [52,53,54,55].

### 3.1. Mitophagy Failure in Cardio-Oncology: PINK1/Parkin Dysfunction, Alternative Mitophagy Pathways, and the Accumulation of Senescence-Inducing Mitochondria

Mitophagy is a selective form of autophagy responsible for removing dysfunctional mitochondria through lysosomal degradation. The best-characterized pathway involves PTEN-induced kinase 1 (PINK1) and the E3 ubiquitin ligase Parkin (PRKN). Under normal mitochondrial conditions, PINK1 is imported into mitochondria and rapidly degraded. However, mitochondrial membrane depolarization stabilizes PINK1 on the outer mitochondrial membrane, allowing phosphorylation of ubiquitin and recruitment of Parkin. Activated Parkin ubiquitinates mitochondrial proteins, facilitating recognition by autophagic adaptors and delivery of damaged mitochondria to lysosomes [56].

In cardiovascular tissues, efficient mitophagy is essential because cardiomyocytes contain exceptionally high mitochondrial density and depend predominantly on oxidative phosphorylation for ATP production. Failure of mitochondrial clearance results in accumulation of ROS-generating mitochondria, increased mitochondrial DNA damage, impaired calcium handling, and energetic insufficiency, all of which contribute to cellular senescence and cardiovascular dysfunction [51,56].

Anthracycline-induced cardiotoxicity represents a clinically relevant example of therapy-associated mitophagy impairment. Doxorubicin initially activates autophagic responses as an adaptive mechanism; however, prolonged exposure disrupts autophagic flux, impairs lysosomal processing, and prevents effective mitochondrial clearance. The resulting accumulation of dysfunctional mitochondria enhances oxidative stress, activates DNA damage pathways, and promotes cardiomyocyte senescence-associated changes [52,53,54,55]. Defective mitophagy is also increasingly recognized in endothelial cells and vascular smooth muscle cells exposed to cardiovascular stressors. Reduced mitochondrial turnover promotes persistence of damaged organelles, amplifies inflammatory signaling, and contributes to endothelial dysfunction and vascular aging [57,58].

Although PINK1/Parkin-mediated mitophagy represents the most extensively studied mitochondrial clearance pathway, several Parkin-independent mechanisms contribute to mitochondrial maintenance. These include receptor-mediated pathways involving BNIP3, BNIP3L/NIX, FUN14 domain-containing protein 1 (FUNDC1), and BCL2-like protein 13 (BCL2L13), which directly interact with LC3/GABARAP proteins to promote mitochondrial elimination [59,60]. Cardiac tissues rely on these alternative pathways because basal mitochondrial turnover must continue under both physiological and pathological conditions. FUNDC1-dependent mitophagy, for example, regulates mitochondrial quality control during hypoxic stress, whereas BNIP3/NIX signaling contributes to mitochondrial remodeling during cardiac adaptation [61,62,63].

Cancer treatments may impair these pathways through oxidative modification of mitophagy proteins, altered mitochondrial membrane potential (MMP), defective lysosomal acidification, and metabolic disturbances. When mitochondrial degradation becomes insufficient, damaged mitochondria remain metabolically active and function as persistent sources of ROS, mitochondrial DNA leakage, and inflammatory signaling [64,65,66]. This mechanism provides a potential explanation for delayed cardiovascular toxicity observed years after completion of cancer therapy. Treatment-induced mitochondrial damage may persist as a form of cellular stress imprint, continuously activating senescence pathways despite removal of the initial therapeutic insult [67,68].

### 3.2. Mitochondrial Dynamics: The Balance Between Fusion, Fission, and Cardiovascular Aging

Mitochondria constantly remodel their morphology through coordinated cycles of fusion and fission. Fusion, mediated primarily by mitofusin-1 (MFN1), mitofusin-2 (MFN2), and optic atrophy protein 1 (OPA1), allows mitochondrial content exchange and preservation of functional networks. Conversely, fission, regulated largely by dynamin-related protein 1 (DRP1), facilitates mitochondrial distribution and segregation of damaged organelles for degradation [41]. Balanced mitochondrial dynamics are essential for cardiovascular homeostasis. Excessive mitochondrial fragmentation increases membrane depolarization, ROS generation, and apoptotic susceptibility, whereas excessive fusion may permit accumulation of damaged mitochondria by preventing efficient organelle segregation and removal [51].

Cancer therapies frequently disturb this equilibrium. Doxorubicin activates DRP1-dependent mitochondrial fragmentation in cardiomyocytes, resulting in impaired oxidative phosphorylation, increased ROS production, and activation of apoptotic and senescence pathways [69,70]. Loss of MFN2 further compromises mitochondrial communication between organelles and the endoplasmic reticulum, affecting calcium regulation and metabolic adaptation [69,70,71].

Radiation exposure also alters mitochondrial morphology by promoting fragmentation, membrane instability, and impaired mitochondrial trafficking. These changes are particularly harmful in endothelial cells, where mitochondrial dynamics regulate angiogenesis, vascular repair, and inflammatory responses [72]. Therefore, mitochondrial dynamics should be viewed not simply as structural remodeling but as a determinant of cellular fate. The balance between fusion, fission, and mitophagy determines whether damaged mitochondria are repaired, eliminated, or retained as drivers of chronic cardiovascular stress [73,74].

### 3.3. Bioenergetic Collapse and Metabolic Reprogramming of Senescent Cardiovascular Cells

Mitochondrial dysfunction fundamentally alters cellular energy metabolism. Healthy cardiomyocytes obtain approximately 70–90% of ATP through mitochondrial oxidative phosphorylation and fatty acid oxidation. Following anticancer therapy-induced mitochondrial injury, respiratory chain activity declines, resulting in reduced ATP production and compensatory metabolic adaptation toward glycolysis [75,76]. Although glycolytic adaptation can temporarily maintain cellular energy requirements, prolonged metabolic remodeling contributes to cardiovascular dysfunction. Reduced mitochondrial ATP availability impairs sarcomere contraction, calcium cycling, ion transport, and endothelial barrier integrity, thereby promoting myocardial dysfunction and vascular abnormalities [75,77].

Senescent cells exhibit characteristic metabolic alterations, including increased glycolytic dependence, altered NAD^+^ metabolism, reduced mitochondrial respiration, and impaired antioxidant capacity [78,79]. These metabolic changes support continued SASP production and inflammatory signaling. Importantly, mitochondrial dysfunction and senescence form a bidirectional relationship. Damaged mitochondria induce senescence through ROS generation and DNA damage, while senescent metabolic remodeling further decreases mitochondrial efficiency and promotes additional mitochondrial injury [80]. This self-reinforcing cycle provides a mechanistic explanation for accelerated cardiovascular aging following cancer therapy.

### 3.4. Mitochondrial Quality Control as a Therapeutic Target in Cardio-Oncology

**Figure 2:**
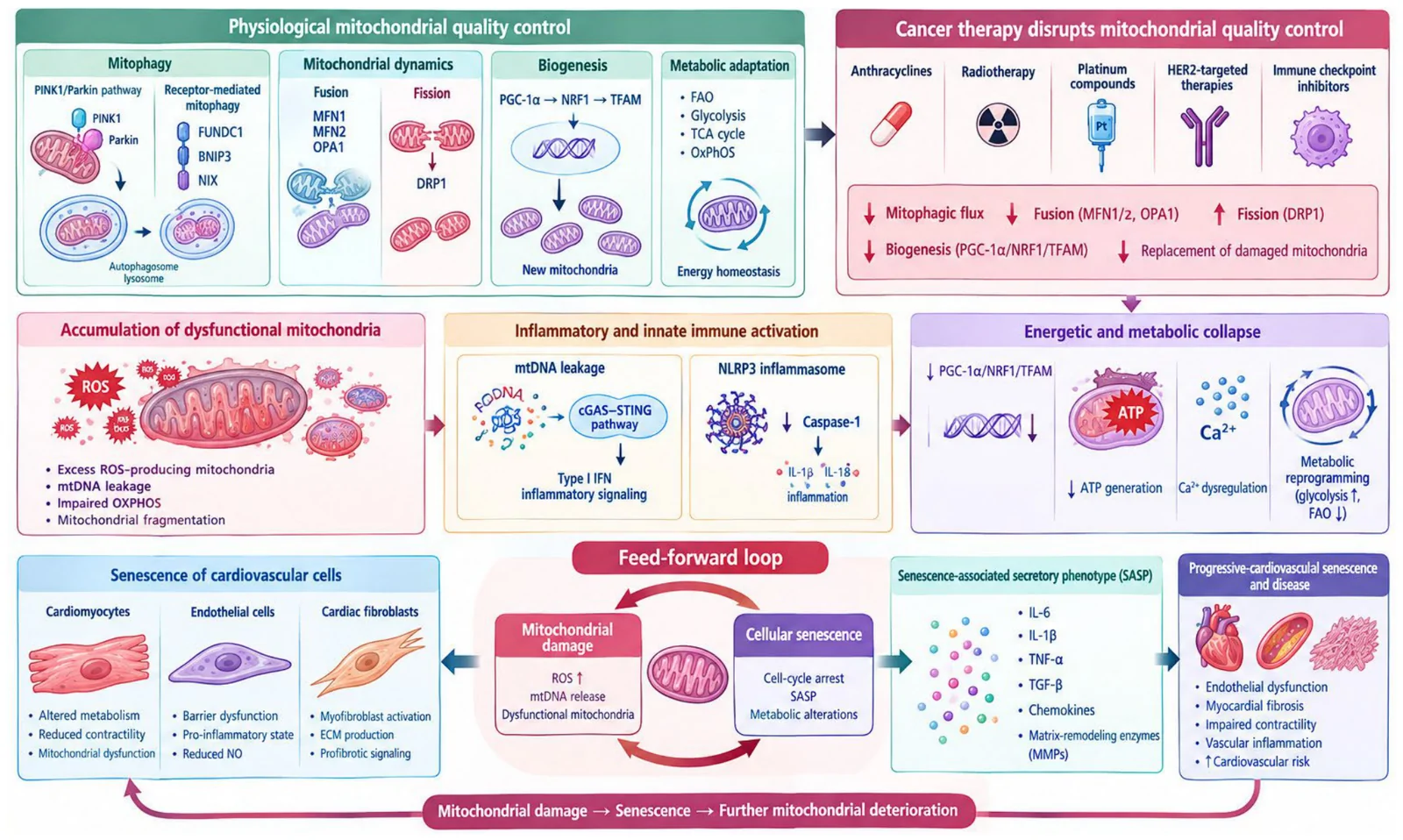
Mitochondrial quality control failure as a mechanistic driver of therapy-induced cardiovascular senescence. Mitochondrial homeostasis depends on coordinated regulation of mitophagy, mitochondrial dynamics, biogenesis, and metabolic adaptation. Under physiological conditions, damaged mitochondria are selectively removed through PINK1/Parkin-dependent and receptor-mediated mitophagy pathways involving FUNDC1, BNIP3, and NIX. Mitochondrial fusion mediated by MFN1, MFN2, and OPA1 promotes functional complementation, whereas DRP1-dependent fission facilitates segregation of damaged mitochondrial components for degradation. Cancer therapies disrupt these quality control systems by impairing mitophagic flux, promoting excessive mitochondrial fragmentation, reducing mitochondrial biogenesis, and limiting replacement of damaged organelles. Defective mitochondrial clearance results in accumulation of ROS-producing mitochondria, mtDNA leakage, activation of cGAS–STING and NLRP3 inflammasome pathways, and persistent inflammatory signaling. Reduced PGC-1α/NRF1/TFAM-mediated mitochondrial renewal further contributes to bioenergetic collapse, impaired ATP generation, calcium dysregulation, and metabolic reprogramming of cardiovascular cells. This mitochondrial quality control failure establishes a feed-forward loop in which mitochondrial damage promotes senescence, while senescence-associated metabolic alterations further accelerate mitochondrial deterioration. Abbreviations: BNIP3, BCL2 interacting protein 3; cGAS, cyclic GMP-AMP synthase; DRP1, dynamin-related protein 1; FUNDC1, FUN14 domain-containing protein 1; MFN, mitofusin; NIX, BNIP3-like protein; NRF1, nuclear respiratory factor 1; OPA1, optic atrophy protein 1; PGC-1α, peroxisome proliferator-activated receptor gamma coactivator 1-alpha; PINK1, PTEN-induced kinase 1; TFAM, mitochondrial transcription factor A.

Restoration of MQC represents a promising therapeutic strategy for preventing cancer therapy-associated cardiovascular disease (Figure 2). Unlike conventional antioxidants, MQC-targeted approaches aim to restore mitochondrial homeostasis by improving mitochondrial turnover, dynamics, and metabolic resilience. Enhancement of mitophagy through activation of PINK1/Parkin, FUNDC1, or BNIP3 pathways may prevent accumulation of dysfunctional mitochondria; however, excessive activation must be avoided because uncontrolled mitochondrial removal can compromise cellular energy production [81,82]. Restoration of mitochondrial biogenesis through activation of PGC-1α, NRF1, NRF2, and TFAM pathways may facilitate replacement of damaged mitochondria and improve cardiovascular metabolic capacity [83,84]. Similarly, modulation of mitochondrial dynamics by regulating DRP1, MFN2, and OPA1 signaling may preserve mitochondrial network integrity and prevent excessive fragmentation [85,86].

Emerging evidence also suggests that MQC-targeted approaches may complement senolytic and senomorphic therapies. Eliminating highly dysfunctional senescent cells while restoring mitochondrial function in remaining cardiovascular cells may represent a future precision strategy for reducing long-term cardiovascular complications in cancer survivors [87,88].

## 4. Cancer Therapy-Specific Mitochondrial Senescence Pathways: Anthracyclines, Radiotherapy, Platinum Compounds, HER2-Targeted Therapy, and Immune Checkpoint Inhibitors

The molecular diversity of anticancer therapies is accompanied by diverse patterns of cardiovascular injury. However, a growing concept in cardio-oncology is that many apparently distinct treatment-associated toxicities converge on common biological processes, including mitochondrial dysfunction, oxidative stress, persistent DNA damage, inflammatory activation, and cellular senescence [1,5]. Cancer therapy-induced cardiovascular senescence provides a mechanistic explanation for delayed cardiovascular complications that persist long after completion of cancer treatment. Unlike acute cardiomyocyte death, senescence establishes a chronic pathological state characterized by stable cell-cycle arrest, mitochondrial remodeling, altered metabolism, and secretion of inflammatory mediators collectively known as the senescence-associated secretory phenotype (SASP) [32,89,90].

Although anthracyclines and radiotherapy remain the most extensively studied triggers of cardiovascular senescence-associated changes, increasing evidence indicates that targeted therapies and immune checkpoint inhibitors also influence mitochondrial homeostasis and senescence-associated pathways [32,89,91,92]. Despite differences in their primary molecular targets, many anticancer treatments ultimately converge on mitochondrial stress responses, suggesting that preservation of mitochondrial resilience may represent a common therapeutic strategy in cardio-oncology.

### 4.1. Anthracycline-Induced Mitochondrial Senescence and Cardiovascular Aging

Anthracyclines, including doxorubicin and daunorubicin, remain essential components of therapy for hematological malignancies and solid tumors. Their clinical efficacy is limited by cumulative dose-dependent cardiovascular toxicity, which may manifest as asymptomatic myocardial dysfunction, cardiomyopathy, arrhythmias, and progressive heart failure [93,94]. Although anthracycline cardiotoxicity was initially attributed primarily to cardiomyocyte apoptosis caused by oxidative injury, recent studies demonstrate that sublethal exposure can induce persistent mitochondrial dysfunction and cellular senescence without extensive cell death [95,96].

#### 4.1.1. Doxorubicin-Induced Mitochondrial ROS and DNA Damage

Doxorubicin accumulates within cardiac mitochondria because of its strong interaction with cardiolipin, resulting in disruption of mitochondrial membrane organization and respiratory chain function. This promotes excessive mitochondrial ROS production, lipid peroxidation, mitochondrial DNA damage, and impairment of oxidative phosphorylation [67,69]. A major mechanism involves interaction between doxorubicin and mitochondrial topoisomerase IIβ (TOP2B), producing DNA double-strand breaks and activating DNA damage signaling pathways. TOP2B-mediated activation of ATM, p53, and downstream p21 signaling contributes to cardiomyocyte growth arrest and senescence-like phenotypes [97]. Concurrent activation of p16^Ink4a^-Rb signaling reinforces irreversible cell-cycle withdrawal and stabilizes the senescent state [14,67,96]. In cardiomyocytes, mitochondrial injury reduces ATP generation, disrupts calcium handling, and promotes contractile dysfunction [19,98,99]. In endothelial cells, doxorubicin-induced oxidative stress reduces nitric oxide bioavailability, impairs angiogenic capacity, and accelerates vascular aging. Senescent cardiovascular cells subsequently release SASP factors, including IL-6, IL-1β, TNF-α, and matrix metalloproteinases, promoting myocardial fibrosis and vascular inflammation [90,100,101].

#### 4.1.2. Anthracycline Effects on Mitochondrial Quality Control

Anthracyclines also disrupt mitochondrial maintenance pathways. Doxorubicin alters mitochondrial dynamics by increasing DRP1-dependent mitochondrial fragmentation while reducing fusion-associated proteins including MFN2 and OPA1 [102,103]. These structural abnormalities impair mitochondrial respiratory efficiency and increase susceptibility to oxidative injury. Furthermore, anthracycline exposure interferes with autophagic flux and mitophagy-mediated mitochondrial clearance, leading to accumulation of damaged mitochondria [104,105]. Failure of mitochondrial quality control creates a reinforcing pathological cycle:

Doxorubicin exposure → mitochondrial injury → impaired mitophagy → ROS accumulation → DNA damage → senescence → SASP-mediated inflammation

This mechanism contributes to delayed cardiotoxicity, explaining why cardiovascular complications may emerge years after apparently successful cancer treatment [90,106,107].

### 4.2. Radiotherapy-Induced Mitochondrial Dysfunction and Cardiovascular Senescence

Radiotherapy is an essential treatment modality for breast, lung, mediastinal, and hematological malignancies. However, radiation exposure involving cardiac structures increases the long-term risk of coronary artery disease, myocardial fibrosis, valvular disease, pericardial abnormalities, and microvascular dysfunction [108,109]. Radiation-induced cardiovascular disease is increasingly recognized as a chronic inflammatory and degenerative process rather than a transient injury. Ionizing radiation generates ROS through water radiolysis and induces persistent mitochondrial abnormalities involving respiratory chain proteins, membrane lipids, and mitochondrial DNA [73,110].

#### Radiation-Induced mtDNA Damage and Senescence Signaling

Mitochondrial DNA is particularly vulnerable to radiation-induced oxidative injury because of its proximity to electron transport chain complexes and limited repair capacity [14,52]. Persistent mtDNA damage reduces mitochondrial transcription, compromises respiratory chain activity, and increases secondary ROS generation. Radiation-induced nuclear and mitochondrial DNA damage activates ATM-dependent DNA damage responses, resulting in p53/p21 and p16/Rb-mediated senescence pathways [111,112]. In endothelial cells, this causes reduced proliferation, impaired angiogenesis, and vascular dysfunction, whereas cardiac fibroblast activation promotes TGF-β-dependent extracellular matrix deposition and myocardial fibrosis [113,114,115,116,117]. Radiation-induced mitochondrial damage also promotes release of oxidized mtDNA into the cytoplasm, activating cGAS–STING signaling and inflammatory cytokine production. This pathway provides a mechanistic connection between radiation exposure, chronic inflammation, SASP activation, and progressive cardiovascular aging [118,119].

### 4.3. Platinum-Based Chemotherapy and Mitochondrial Stress-Induced Senescence

Platinum compounds, including cisplatin and carboplatin, are widely used against testicular, ovarian, lung, bladder, and head-and-neck cancers. Although nephrotoxicity and neurotoxicity are recognized adverse effects, increasing evidence indicates that platinum agents contribute to cardiovascular injury through oxidative and mitochondrial mechanisms [120,121]. Cisplatin forms DNA adducts in both nuclear and mitochondrial genomes, impairing replication and transcription. Because mitochondrial DNA lacks extensive protective histone packaging, it is highly vulnerable to platinum-induced damage. Accumulation of mtDNA lesions disrupts oxidative phosphorylation and enhances mitochondrial ROS generation [122,123].

Mitochondrial oxidative stress activates p53-dependent DNA damage responses and promotes senescence-associated inflammatory signaling [124]. In endothelial cells, platinum exposure decreases nitric oxide production, increases oxidative stress, and reduces vascular repair capacity, thereby contributing to endothelial dysfunction and accelerated vascular aging [125,126]. Furthermore, platinum-associated mitochondrial injury in endothelial progenitor cells may impair vascular regeneration and promote long-term cardiovascular vulnerability among cancer survivors [127,128,129].

Furthermore, cisplatin is the most extensively studied with respect to mitochondrial-mediated cardiotoxicity among platinum-based chemotherapeutic agents, whereas oxaliplatin has comparatively lesser direct evidence of cardiac mitochondrial injury. Cisplatin favorably targets mtDNA, forming platinum–mtDNA complexes that impair mtDNA replication and transcription, thereby hampering the synthesis of ETC proteins and disrupting oxidative phosphorylation, ultimately leading to ATP depletion [130,131,132,133]. Moreover, cisplatin directly damages the activity of ETC complexes I, III, and IV, resulting in bioenergetic failure and excessive mitochondrial mtROS production, which enhances oxidative damage to lipids, proteins, and nucleic acids.

Mitochondrial dysfunction is further exacerbated by the loss of MMP and increased opening of the mitochondrial permeability transition pore (mPTP), enabling cytochrome c release, caspase activation, and intrinsic cardiomyocyte apoptosis. In parallel, cisplatin deteriorates mitochondrial antioxidant defenses through depletion of glutathione (GSH) and reduction of antioxidant enzymes, including superoxide dismutase (SOD), glutathione peroxidase (GPx), and catalase, thereby amplifying oxidative stress and mitochondrial injury [134,135].

Emerging evidence also suggests that cisplatin disrupts MQC by promoting disproportionate Drp1-mediated mitochondrial fission, decreasing the expression of fusion proteins MFN2 and OPA1, and obstructing PINK1/Parkin-dependent mitophagy, leading to the accumulation of impaired mitochondria and further deterioration of cardiac function. In parallel, although oxaliplatin has been shown to induce mtDNA damage, ETC dysfunction, mitochondrial ROS generation, membrane depolarization, and apoptotic signaling in experimental systems, evidence directly linking these mechanisms to cardiotoxicity remains relatively sparse compared with cisplatin [136,137,138].

### 4.4. HER2-Targeted Therapy: Mitochondrial Dysfunction Through Survival Signaling Disruption

HER2-targeted therapies, particularly trastuzumab, have substantially improved outcomes in HER2-positive breast cancer. However, HER2 signaling also contributes to cardiomyocyte survival, stress adaptation, and mitochondrial metabolic regulation [139,140]. Unlike anthracyclines, HER2 inhibitors generally cause myocardial dysfunction without extensive cardiomyocyte apoptosis. Instead, they disrupt neuregulin-1/HER2/ErbB signaling, reducing PI3K–Akt pathway activation required for mitochondrial maintenance, antioxidant defense, and cellular adaptation [141,142]. Reduced Akt signaling impairs metabolic regulation and decreases mitochondrial stress tolerance, resulting in increased ROS production, impaired ATP generation, and enhanced susceptibility to senescence. The combination of anthracycline exposure followed by trastuzumab is particularly harmful because anthracyclines generate mitochondrial injury while HER2 blockade reduces the capacity for mitochondrial repair and adaptation [141,143]. This therapeutic interaction illustrates how sequential anticancer therapies can synergistically accelerate cardiovascular biological aging.

According to recent transcriptomic and experimental studies, HER2 suppression extends beyond autophagy to regulate several components of mitochondrial quality control. For example, trastuzumab alters the expression of genes involved in mitochondrial biogenesis, mitochondrial organization, autophagy, and mitophagy, indicating a broad disruption of mitochondrial homeostasis in cardiomyocytes [144].

In addition, trastuzumab inhibits AMPK signaling, causing reduced PGC-1α, NRF1, and TFAM activity, which abrogates mitochondrial biogenesis, decreases ETC protein expression, depresses ATP-linked respiration, and impairs metabolic flexibility. Activation of the AMPK–PGC-1α–SIRT3 axis reestablishes MMP, enhances autophagic flux, improves mitochondrial respiratory function, and partially salvages trastuzumab-induced cardiomyocyte injury, highlighting this pathway as a promising therapeutic target [145]. Collectively, these findings indicate that trastuzumab-incited cardiotoxicity results not only from inhibition of HER2-facilitated survival signaling but also from disruption of multiple mitochondrial quality control pathways-including autophagy/mitophagy, mitochondrial biogenesis, redox homeostasis, and cellular bioenergetics-whereas comparable mechanistic evidence for pertuzumab remains limited [146].

### 4.5. Immune Checkpoint Inhibitors: Mitochondrial Inflammation and Senescence-Associated Cardiac Injury

Immune checkpoint inhibitors (ICIs), including PD-1, PD-L1, and CTLA-4 inhibitors, have transformed treatment of melanoma, lung cancer, renal cancer, and other malignancies. However, immune activation can produce cardiovascular complications including myocarditis, vasculitis, arrhythmias, and inflammatory cardiomyopathy [147,148,149,150]. Unlike conventional chemotherapy, ICI-associated cardiovascular injury is primarily immune-mediated. Nevertheless, mitochondria remain central regulators of immune metabolism and inflammatory amplification. Activated immune cells undergo metabolic remodeling with increased mitochondrial activity and ROS production during effector responses [151,152].

In ICI-associated myocarditis, activated T cells infiltrate cardiac tissue and generate inflammatory cytokines, resulting in myocardial injury. Damaged cardiomyocytes release mitochondrial danger signals, including mtDNA and cardiolipin, activating cGAS–STING and NLRP3 inflammasome pathways [118,153,154,155,156]. Although immune checkpoint inhibitor (ICI)-associated myocarditis is a well-established acute immune-mediated cardiovascular toxicity supported by clinical and pathological evidence [157,158], the mechanistic links between ICI exposure, persistent mitochondrial dysfunction, cellular senescence, and long-term cardiovascular remodeling are currently supported predominantly by experimental studies and remain incompletely validated in patients [158,159]. Preclinical investigations suggest that sustained mitochondrial injury, excessive mitochondrial reactive oxygen species (mtROS) production, and chronic inflammatory signaling may contribute to senescence-associated cardiovascular alterations following ICI therapy; however, direct clinical evidence demonstrating a causal relationship between ICI treatment and chronic cardiovascular aging is still limited [160]. Therefore, in this review, acute ICI-associated myocarditis is distinguished from the proposed mechanisms underlying delayed cardiovascular sequelae, and speculative concepts are clearly identified where definitive clinical validation is lacking.

## 5. Integrated Model: Convergence of Cancer Therapies on Mitochondrial Senescence Pathways

Despite distinct pharmacological mechanisms, major anticancer therapies converge on mitochondrial pathways regulating cardiovascular aging.

Collectively, these observations support mitochondria as a central biological interface connecting cancer therapy exposure with premature cardiovascular aging (Table 1). Therapeutic approaches aimed at preserving mitochondrial quality control, reducing maladaptive ROS signaling, and preventing senescent cell accumulation may therefore provide broad cardioprotective benefits across multiple cancer treatment modalities.

## 6. Biomarkers, Therapeutics, and Future Perspectives: Translating Mitochondrial Senescence Biology into Precision Cardio-Oncology

The recognition of mitochondrial dysfunction and cellular senescence as central mechanisms linking cancer therapy with cardiovascular disease has created new opportunities for early detection, risk stratification, and targeted intervention. Current clinical surveillance strategies primarily rely on conventional measures of cardiac injury and dysfunction, including cardiac troponins, natriuretic peptides, echocardiographic parameters, and global longitudinal strain. Although these approaches have improved recognition of overt cancer therapy-related cardiovascular toxicity (CTR-CVT), they frequently identify injury after irreversible molecular remodeling has already occurred. Emerging evidence suggests that mitochondrial stress, senescence-associated signaling, and inflammatory biomarkers may provide earlier indicators of cardiovascular vulnerability, enabling preventive rather than reactive management. MicroRNAs (miRNAs) have emerged as important post-transcriptional regulators of mitochondrial quality control (MQC) and are increasingly recognized as contributors to cancer therapy-induced cardiovascular senescence.

The clinical translation of mitochondria-centered cardio-oncology requires integration of molecular biomarkers, advanced imaging, functional assessment, and individualized therapeutic approaches. Importantly, interventions must preserve anticancer efficacy while preventing accelerated cardiovascular aging. This balance represents one of the major challenges in developing senescence-targeted strategies for cancer survivors.

### 6.1. Biomarkers of Mitochondrial Dysfunction and Cardiovascular Senescence

#### 6.1.1. Conventional Cardiac Injury Biomarkers

Cardiac troponins (particularly high-sensitivity cardiac troponin I and T) remain among the most clinically validated biomarkers for detecting myocardial injury during cancer therapy. Increased troponin concentrations after anthracycline administration, immune checkpoint inhibitor therapy, or other cardiotoxic treatments correlate with subsequent reductions in left ventricular function and increased cardiovascular risk [161,162,163]. Similarly, natriuretic peptides, including B-type natriuretic peptide (BNP) and N-terminal pro-BNP (NT-proBNP), reflect myocardial wall stress and neurohormonal activation. Although useful for identifying established cardiac dysfunction, these markers primarily represent downstream consequences rather than the initiating mechanisms of mitochondrial injury [163]. Therefore, while conventional biomarkers remain essential components of cardio-oncology monitoring, they have limited ability to detect early mitochondrial stress and therapy-induced senescence.

#### 6.1.2. Mitochondrial-Derived Biomarkers

##### Circulating Mitochondrial DNA (mtDNA)

Mitochondrial DNA released into circulation represents a promising biomarker of mitochondrial injury and sterile inflammation. Damaged mitochondria release fragmented or oxidized mtDNA, which functions as a danger-associated molecular pattern (DAMP) capable of activating cGAS–STING signaling and innate immune responses [164,165]. Elevated circulating cell-free mtDNA has been detected in conditions associated with oxidative stress, inflammation, aging, and cardiovascular disease. In cancer survivors, increased mtDNA release may reflect persistent mitochondrial injury induced by chemotherapy or radiotherapy and may serve as an early indicator of cardiovascular biological aging [128,164,166]. However, clinical application remains limited by methodological variability, including differences in DNA extraction techniques, mitochondrial genomic targets, and normalization strategies. In particular, platelet contamination, variability in separation of extracellular vesicle-associated versus free mtDNA, and pre-analytical handling can markedly influence measured circulating cell free-mtDNA concentrations. Quantification methods based mainly on quantitative PCR (qPCR) also necessitate careful assay optimization because dissimilarities in primer selection, amplification efficiency, mitochondrial genomic regions targeted, and potential interference from nuclear mitochondrial DNA segments may introduce measurement bias. Therefore, standardized protocols incorporating validated reference materials, appropriate normalization strategies (e.g., parallel measurement of cell free-mtDNA and cell free nuclear-DNA, assay validation parameters such as limit of detection, limit of quantification, specificity, and inter-laboratory reproducibility are essential before cell free-mtDNA can be implemented as a reliable biomarker for cancer therapy-related cardiovascular toxicity and mitochondrial dysfunction [167,168,169].

##### Mitochondrial ROS and Oxidative Stress Markers

Because mitochondrial ROS represents a central driver of therapy-induced cardiovascular senescence, oxidative stress biomarkers have significant translational potential. Candidate markers include: 8-hydroxy-2′-deoxyguanosine (8-OHdG), reflecting oxidative DNA damage, oxidized glutathione (GSSG), lipid peroxidation products such as malondialdehyde and 4-hydroxynonenal, oxidized proteins and mitochondrial antioxidant enzyme alterations. These markers may identify patients with increased susceptibility to mitochondrial injury before development of clinical cardiotoxicity [170,171].

#### 6.1.3. Senescence-Associated Biomarkers

Reliable identification of senescent cardiovascular cells remains challenging because no single marker uniquely defines cellular senescence. Current approaches rely on combinations of molecular features including: cell-cycle arrest markers [p16^Ink4a^, p21^Cip1^, hypophosphorylated retinoblastoma protein (Rb)], senescence-associated phenotypes [senescence-associated β-galactosidase (SA-β-gal), senescence-associated heterochromatin foci (SAHF)], reduced lamin B1 expression, SASP-associated factors (IL-6, IL-1β, TNF-α, MCP-1, TGF-β, matrix metalloproteinases). In cancer survivors, circulating SASP factors and senescence-related proteins have been associated with accelerated biological aging and cardiovascular risk [3,172,173].

#### 6.1.4. MicroRNAs and Multi-Omic Biomarkers

MicroRNAs (miRNAs) represent another promising class of biomarkers because they regulate mitochondrial metabolism, inflammation, fibrosis, and cellular senescence. By targeting key components of mitochondrial biogenesis, including the PGC-1α–NRF1/2–TFAM axis, specific miRNAs can suppress mitochondrial renewal and impair oxidative phosphorylation [174,175]. Other miRNAs regulate mitochondrial dynamics by modulating the expression of DRP1, MFN1/2, and OPA1, thereby influencing the balance between mitochondrial fission and fusion, while additional miRNAs affect PINK1/Parkin-mediated mitophagy, leading to the accumulation of dysfunctional mitochondria [176,177]. Furthermore, dysregulated miRNA expression contributes to excessive mitochondrial reactive oxygen species (mtROS) production, activation of redox-sensitive signaling pathways, and amplification of inflammatory responses, including NF-κB- and NLRP3-mediated signaling, thereby promoting senescence-associated secretory phenotype (SASP) development and persistent cardiovascular dysfunction following cancer therapy [47,178,179].

Examples include: miR-34a (senescence activation and mitochondrial dysfunction), miR-21 (fibrosis and inflammatory remodeling), miR-126 (endothelial integrity and vascular repair) [180,181,182]. Integration of circulating miRNA profiles with proteomic, metabolomic, and genomic datasets may enable personalized prediction of cardiotoxic risk [183]. Although direct evidence linking specific miRNAs to cancer therapy-induced cardiovascular senescence remains inadequate, accruing studies indicate that miRNA-mediated regulation of mitochondrial dysfunction, oxidative stress, and inflammatory signaling may represent an important mechanistic layer contributing to this process. Future biomarker development will likely depend on multi-omic approaches combining mitochondrial signatures, inflammatory markers, imaging parameters, and patient-specific genetic susceptibility.

### 6.2. Therapeutic Strategies Targeting Mitochondrial Senescence

#### 6.2.1. Mitochondria-Targeted Antioxidants

Traditional antioxidant supplementation has shown inconsistent results because nonspecific ROS suppression may interfere with physiological signaling and anticancer mechanisms. Therefore, current approaches focus on mitochondria-directed antioxidants capable of selectively reducing pathological mitochondrial oxidative stress. Examples include: Mitoquinone (MitoQ) which combines ubiquinone with a lipophilic triphenylphosphonium cation, enabling mitochondrial accumulation. Experimental studies demonstrate protection against oxidative mitochondrial damage, endothelial dysfunction, and cardiac remodeling [184,185]. SS Peptides (Szeto–Schiller peptides) selectively accumulate within mitochondria and stabilize cardiolipin, reducing ROS generation and preserving mitochondrial membrane integrity [186,187,188]. Although promising, clinical validation in cancer survivors remains incomplete.

#### 6.2.2. Restoration of Mitochondrial Quality Control

Because defective mitochondrial clearance contributes strongly to senescence, restoration of mitophagy represents an attractive therapeutic approach. Potential strategies include: activation of PINK1/Parkin signaling, enhancement of FUNDC1-mediated mitophagy, restoration of lysosomal function, and activation of mitochondrial biogenesis pathways. Agents that stimulate AMPK, SIRT1, or PGC-1α pathways may improve mitochondrial renewal and metabolic flexibility [8,189,190]. However, excessive activation of mitophagy could impair energy availability, emphasizing the need for precise therapeutic modulation [191,192,193]. Recent studies have elucidated the Rho/MRTF/SRF signaling axis as an imperative regulator of mitochondrial homeostasis, extending the role of serum response factor (SRF) beyond its established functions in cardiac development, hypertrophy, fibrosis, and cancer progression. Pharmacological inhibition of the Rho/MRTF/SRF pathway using small-molecule inhibitors such as CCG-1423, CCG-203971, and CCG-232601 has been shown to alter the expression of genes governing mitochondrial biogenesis, dynamics, and bioenergetics. These inhibitors suppress oxidative phosphorylation, decrease mitochondrial ATP production, impede ETC activity, enhance glycolytic dependence, and perturb mitochondrial dynamics, signifying that SRF signaling is closely linked to mitochondrial metabolic regulation [194,195]. Conversely, cardiac-specific SRF overexpression induces profound mitochondrial abnormalities, including reduced expression of PGC-1α, PGC-1β, and OPA1, increased DRP1 expression, impaired oxidative phosphorylation, increased oxidative stress, disrupted calcium homeostasis, and accelerated cardiac aging, supporting a direct role for SRF in maintaining mitochondrial integrity [196]. Collectively, these findings advocate that dysregulated SRF signaling contributes to mitochondrial dysfunction, excessive mitochondrial ROS generation, abrogated mitochondrial dynamics, and defective bioenergetics—hallmarks that are increasingly recognized as drivers of cellular senescence and cardiovascular injury. Given that cancer therapies frequently disrupt mitochondrial quality control, modulation of the Rho/MRTF/SRF pathway may represent a promising strategy for preserving mitochondrial homeostasis and mitigating therapy-induced cardiovascular senescence, although its therapeutic relevance in cancer survivors remains to be established in preclinical and clinical studies.

#### 6.2.3. Senolytic and Senomorphic Therapies

Senolytics selectively eliminate senescent cells, whereas senomorphic agents suppress harmful SASP signaling without removing senescent cells. Major candidate senolytics include: Dasatinib, Quercetin, Fisetin, and Bcl-2 family inhibitors [197,198,199]. Preclinical studies demonstrate that removal of senescent cells can improve vascular function, reduce inflammation, and enhance tissue regeneration [199,200,201]. Senomorphic approaches aim to suppress SASP production through modulation of: NF-κB signaling, mTOR pathways, and inflammatory cytokine networks. Potential senomorphic agents include: rapamycin, metformin, NAD^+^ precursors, and anti-inflammatory pathway inhibitors. These strategies may be particularly attractive because complete elimination of senescent cells could interfere with beneficial tumor-suppressive functions [202,203].

#### 6.2.4. Cardioprotective Pharmacological Strategies

Several established cardiovascular medications demonstrate potential mitochondrial protective effects.

##### Dexrazoxane

Dexrazoxane remains the only clinically approved cardioprotective agent for reducing anthracycline-associated cardiotoxicity. Its protective effects involve iron chelation and reduction of oxidative injury [204,205].

##### ACE Inhibitors and β-Blockers

Neurohormonal blockade with ACE inhibitors and β-blockers has demonstrated benefit in selected high-risk cancer patients by reducing ventricular remodeling and improving cardiac function [206,207,208].

##### SGLT2 Inhibitors

Emerging evidence suggests SGLT2 inhibitors may preserve mitochondrial function, reduce oxidative stress, and improve metabolic flexibility, although dedicated cardio-oncology trials remain needed [209,210,211].

### 6.3. Future Perspectives and Unresolved Challenges

#### 6.3.1. Development of Precision Cardio-Oncology

Future management of cancer therapy-induced cardiovascular aging will likely move from generalized monitoring toward personalized risk prediction. Important considerations include: genetic susceptibility, mitochondrial haplotypes, baseline biological age, metabolic status, inflammatory profile, and cumulative treatment exposure. Integration of artificial intelligence with multi-omic datasets may enable individualized prediction of cardiovascular risk before clinical disease develops.

#### 6.3.2. Timing and Safety of Senescence-Targeted Therapy

Several important questions remain unresolved:

(a) When should senescence-targeted therapies be initiated?

Early intervention may prevent irreversible remodeling, but premature suppression of senescence may interfere with normal tissue repair.

(b) How can cardiovascular protection avoid reducing anticancer efficacy?

Some senescence pathways contribute to tumor suppression, requiring careful therapeutic selection.

(c) Which patients benefit most?

Biomarker-driven selection will likely be essential to identify individuals with persistent mitochondrial dysfunction and high cardiovascular risk.

#### 6.3.3. Integration of Mitochondrial Biology into Clinical Practice

Future cardio-oncology programs may incorporate: circulating mtDNA analysis, mitochondrial metabolic profiling, senescence-associated biomarkers, advanced cardiac imaging, and digital monitoring technologies. A comprehensive “mitochondrial cardiovascular aging score” combining molecular and clinical parameters could provide a future framework for precision surveillance.

### 6.4. Conclusions and Future Directions

**Figure 3:**
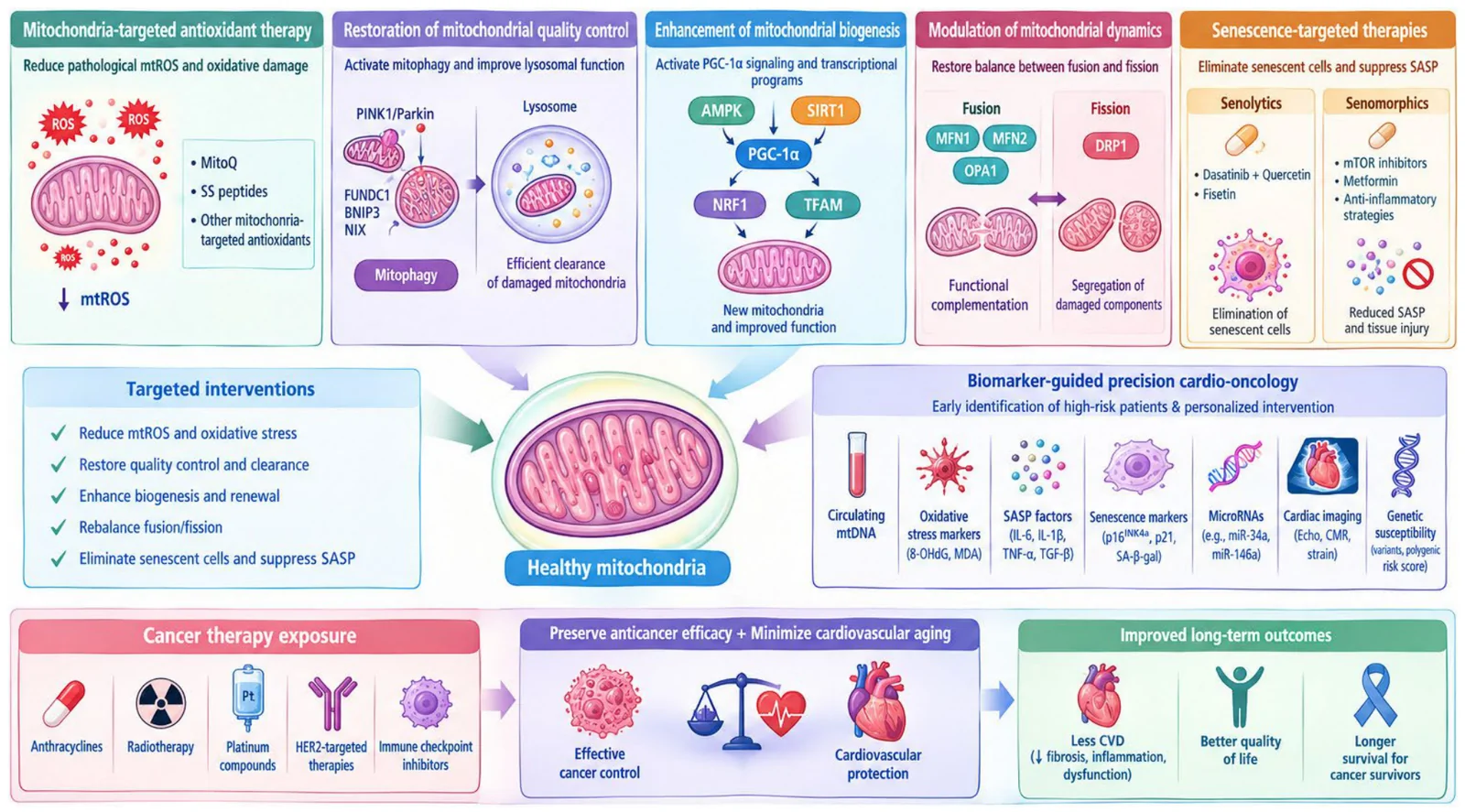
Mitochondria-targeted therapeutic strategies for prevention and reversal of cancer therapy-associated cardiovascular senescence. Therapeutic approaches targeting mitochondrial dysfunction represent a promising strategy for reducing long-term cardiovascular complications among cancer survivors. Potential interventions include mitochondria-directed antioxidants (MitoQ, SS peptides, and related compounds) to reduce pathological mtROS production; restoration of mitochondrial quality control through activation of mitophagy pathways and improvement of lysosomal function; enhancement of mitochondrial biogenesis through PGC-1α, AMPK, SIRT1, NRF1, and TFAM signaling; and modulation of mitochondrial dynamics to restore appropriate balance between fusion and fission. Senescence-targeted approaches include senolytic therapies (such as dasatinib/quercetin and fisetin) to eliminate highly dysfunctional senescent cells and senomorphic therapies (including mTOR inhibitors, metformin, and anti-inflammatory strategies) to suppress SASP-mediated tissue injury. Biomarker-guided precision cardio-oncology approaches integrating circulating mtDNA, oxidative stress markers, SASP factors, senescence markers (p16^Ink4a^, p21^Cip1^, SA-β-gal), microRNAs, cardiac imaging parameters, and genetic susceptibility profiles may enable early identification of high-risk patients and individualized cardioprotective interventions. The proposed framework emphasizes maintaining anticancer efficacy while minimizing therapy-associated mitochondrial aging and cardiovascular disease. Abbreviations: AMPK, AMP-activated protein kinase; mtDNA, mitochondrial DNA; mtROS, mitochondrial reactive oxygen species; mTOR, mechanistic target of rapamycin; SA-β-gal, senescence-associated β-galactosidase; SASP, senescence-associated secretory phenotype; SIRT1, sirtuin 1.

Cancer therapy-associated cardiovascular disease represents a form of accelerated biological aging driven by persistent mitochondrial dysfunction, cellular senescence, and inflammatory remodeling. Mitochondria function as central regulators linking anticancer therapy exposure to oxidative stress, DNA damage, metabolic decline, and SASP activation. Future progress requires multidisciplinary integration of molecular cardiology, oncology, mitochondrial biology, and precision medicine (Figure 3). Biomarkers capable of detecting mitochondrial stress and senescence before irreversible cardiovascular damage occurs will be essential for preventive intervention. Therapeutic approaches combining mitochondrial protection, senescence modulation, and conventional cardiovascular therapies may ultimately allow cancer survivors to achieve longer survival without the burden of premature cardiovascular aging.

Future clinical translation of mitochondria-targeted strategies in cancer survivors will require well-designed prospective clinical trials that evaluate their efficacy, safety, and long-term cardiovascular benefits. Although preclinical studies support interventions targeting mitochondrial quality control, oxidative stress, and bioenergetic dysfunction, their therapeutic potential remains largely unvalidated in human cancer populations. Such trials should incorporate longitudinal assessment of mitochondrial biomarkers, cardiac imaging parameters, and functional outcomes to identify patients who may benefit most from mitochondria-directed therapies. Establishing standardized endpoints and integrating cardiology–oncology collaborative approaches will be essential for advancing precision strategies to prevent or mitigate late cardiovascular complications after cancer therapy.

## Figures and Tables

**Table 1:** Cancer therapy associated mitochondrial senescence pathways.

Therapy	Major Mitochondrial Alteration	Senescence Mechanism
Anthracyclines	Cardiolipin binding, TOP2B damage, ROS generation, impaired mitophagy	ATM–p53/p21 and p16/Rb activation, SASP
Radiotherapy	mtDNA oxidation, respiratory dysfunction, cGAS–STING activation	DNA damage-induced senescence and inflammation
Platinum compounds	mtDNA adduct formation, oxidative stress	ROS-dependent DDR activation
HER2 inhibitors	Loss of survival signaling and mitochondrial adaptation	Metabolic stress-associated senescence
Immune checkpoint inhibitors	Immune-mediated mitochondrial inflammation	mtDAMP-driven inflammatory remodeling

## Data Availability

No new data were created or analyzed in this study. Data sharing is not applicable to this article.
